# Oxic and Anoxic Organic Polymer Degradation Potential of Endophytic Fungi From the Marine Macroalga, *Ecklonia radiata*

**DOI:** 10.3389/fmicb.2021.726138

**Published:** 2021-10-18

**Authors:** Anita K. Perkins, Andrew L. Rose, Hans-Peter Grossart, Keilor Rojas-Jimenez, Selva K. Barroso Prescott, Joanne M. Oakes

**Affiliations:** ^1^Centre for Coastal Biogeochemistry, Faculty of Science and Engineering, Southern Cross University, Lismore, NSW, Australia; ^2^Southern Cross Geoscience, Faculty of Science and Engineering, Southern Cross University, Lismore, NSW, Australia; ^3^Leibniz Institute for Freshwater Ecology and Inland Fisheries (IGB), Experimental Limnology, Berlin, Germany; ^4^Institute of Biochemistry and Biology, University of Potsdam, Potsdam, Germany; ^5^Escuela de Biologia, Universidad de Costa Rica, San Jose, Costa Rica; ^6^National Marine Science Centre, Faculty of Science and Engineering, Southern Cross University, Coffs Harbour, NSW, Australia

**Keywords:** kelp, fungi, endophytes, carbon cycling, extracellular enzymes, cellulose, polymeric organic matter

## Abstract

Cellulose and chitin are the most abundant polymeric, organic carbon source globally. Thus, microbes degrading these polymers significantly influence global carbon cycling and greenhouse gas production. Fungi are recognized as important for cellulose decomposition in terrestrial environments, but are far less studied in marine environments, where bacterial organic matter degradation pathways tend to receive more attention. In this study, we investigated the potential of fungi to degrade kelp detritus, which is a major source of cellulose in marine systems. Given that kelp detritus can be transported considerable distances in the marine environment, we were specifically interested in the capability of endophytic fungi, which are transported with detritus, to ultimately contribute to kelp detritus degradation. We isolated 10 species and two strains of endophytic fungi from the kelp *Ecklonia radiata*. We then used a dye decolorization assay to assess their ability to degrade organic polymers (lignin, cellulose, and hemicellulose) under both oxic and anoxic conditions and compared their degradation ability with common terrestrial fungi. Under oxic conditions, there was evidence that Ascomycota isolates produced cellulose-degrading extracellular enzymes (associated with manganese peroxidase and sulfur-containing lignin peroxidase), while Mucoromycota isolates appeared to produce both lignin and cellulose-degrading extracellular enzymes, and all Basidiomycota isolates produced lignin-degrading enzymes (associated with laccase and lignin peroxidase). Under anoxic conditions, only three kelp endophytes degraded cellulose. We concluded that kelp fungal endophytes can contribute to cellulose degradation in both oxic and anoxic environments. Thus, endophytic kelp fungi may play a significant role in marine carbon cycling via polymeric organic matter degradation.

## Introduction

Coastal habitats have been recognized as carbon (C) cycling hotspots with the potential to influence climate through organic matter (OM) decomposition (Wang and D’Odorico, 2013) controlling the balance between atmospheric CO_2_ and O_2_ (Guenet et al., 2010). Coastal vegetation, including mangroves, seagrasses, salt marshes, and macroalgae supply a significant portion of polymeric, organic C (lignin, cellulose, and hemicellulose) to coastal sediments (Duarte et al., 2005; McLeod et al., 2011; Ortega et al., 2020). Furthermore, macroalgae form the largest vegetated habitats in the coastal ocean (ca. 3.4 million km^2^) with a total annual primary production of ∼ 1.5 Pg C y^–1^ (Krause-Jensen and Duarte, 2016; Krause-Jensen et al., 2018; Raven, 2018). A significant amount (82%) of macroalgae biomass enters the detrital pathway (Krumhansl and Scheibling, 2012), forming an essential component of the marine C cycle. Of all macroalgae, brown macroalgae (kelp) deposit the largest quantities of detritus, globally, in a variety of receiver habitats from sandy beaches to the deep ocean (Krause-Jensen and Duarte, 2016). This detritus is predominantly comprised of cellulose, hemicellulose (mannan, xylan) and sulfated polysaccharides (carrageenan) (Trevathan-Tackett et al., 2015; Deniaud-Bouët et al., 2017), which may ultimately be buried in the sediment, leached into groundwater (Queirós et al., 2019), or degraded through biological processes (Wang and D’Odorico, 2013). Whether kelp detritus is stored as blue carbon or remineralized depends mainly on microbial communities (Raghukumar, 2004), temperature (Passerini et al., 2016), and oxygen concentration; these are fundamental variables controlling biogeochemical processes (Glud, 2008). Degradation pathways can have a significant influence on coastal and marine C cycling, and the global C budget (Guenet et al., 2010; Worden et al., 2015; Jeewon et al., 2017; Grossart et al., 2019; Chrismas and Cunliffe, 2020). Although the role of bacteria in marine detritus degradation is reasonably well known, the potential role of marine fungi has received relatively little attention.

In terrestrial systems, research has shown that fungi (Ascomycota and Basidiomycota) are the major microbial organisms responsible for degradation of refractory polymers (lignin, cellulose, and chitin) (Van der Wal et al., 2013; Berlemont, 2017; Amend et al., 2019; Weinberger et al., 2020). Fungi are known to occur in marine (Hyde et al., 1998; Bonugli-Santos et al., 2015; Behera et al., 2017), and coastal environments (Alsheikh-Hussain et al., 2014; Lakshmi et al., 2017; Amend et al., 2019), however, studies in marine systems have so far mainly focused on characterizing the fungal community rather than assessing its function (Grossart et al., 2019). The ocean floor is a key marine habitat receiving a substantial fraction of the sinking marine detritus that could be degraded by fungi. However large sections of the ocean floor and deeper coastal sediments (below the upper few centimeters of sediment) are anoxic (Watson, 2016) and the potential for fungal degradation to occur in anoxic sediments has been recognized only since the past decade (Ivarsson et al., 2016; Drake and Ivarsson, 2018; Zain UI Arifeen et al., 2019). Marine macroalgae, which are a major contributor of marine detritus, provide an excellent habitat for endophytic fungi, where fungi live in symbiosis within the macroalgae tissues (Suryanarayanan et al., 2010; Suryanarayanan, 2012). Once the macroalgae dies, these endophytic fungi have the ability to turn in to saprophytes and degrade the dead macroalgae biomass (Hyde and Soytong, 2008; Balabanova et al., 2018). Thus, endophytic fungi may be transported within the macroalgae tissue over long distances and become ultimately buried within deep marine sediments or in coastal sediments. This gives fungi the opportunity to remineralize the detritus to a large extent before colonization by any other decomposer, and the ability to potentially influence colonization by other decomposers (Cline et al., 2018; Wolfe and Ballhorn, 2020). Endophytic fungi may therefore directly and indirectly affect degradation of macroalgae detritus.

Fungi produce a range of versatile enzymes to degrade refractory, polymeric compounds such as lignin, chitin, cellulose, hemicellulose, and phenolics (Harms et al., 2011; Wang and D’Odorico, 2013; Khatoon et al., 2017), some of which are found in macroalgae detritus. The primary fungal degradation enzymes are laccases (Janusz et al., 2017), manganese-dependent peroxidase (MnP), manganese-independent peroxidase (MiP), lignin peroxidase (LiP) (Hammel, 1997), and protocatechuate-3,4 dioxygenase (Leonowicz et al., 2001). These enzymes are assisted by secondary enzymes (aryl alcohol oxidase, and glyoxal oxidase) which produce H_2_O_2_ and ⋅OH (Shao et al., 2020). A third group of enzymes [glucose oxidase and cellobiose dehydrogenase (quinone oxidoreductase)] play a crucial role in degrading high-molecular-weight woody material, acting as a feedback system for the primary and secondary enzymes. While these enzymes work synergistically to enhance microbial degradation (Wood and Garcia-Campayo, 1990; Chen et al., 2021), certain enzymes such as laccases, because of their multi-copper oxidases, can also act separately to break down lignin (Leonowicz et al., 2001). Fungi can also potentially break down organic C by inducing cellulose degradation via short-lived reactive oxygen species (ROS), including hydroxyl radicals (⋅OH), superoxide anion radicals (O_2_^–^⋅), and singlet oxygen (^1^O_2_) formation (Ślesak et al., 2019; Smirnoff and Arnaud, 2019). The short-lived ROS are often formed from the longer-lived ROS H_2_O_2_, which is commonly produced in eukaryotic mitochondria (Ivarsson et al., 2016). Hydroxyl radicals and singlet oxygen, in particular, are potent oxidants that can degrade a variety of polymeric, organic C compounds and whose activity can be catalyzed by the presence of other redox-active compounds, such as Cu, Fe, or specific organic moieties (Rose et al., 2010). These same degradation pathways are known to exist in marine and terrestrial fungi, but the knowledge of their ability to degrade polymers in anoxic marine sediments can be improved.

Fungal polymer degradation under oxic conditions has been well studied, where oxygen is used as a terminal electron acceptor (Canfield et al., 2005). Fungal degradation under anoxic conditions is less well known, although yeasts are active under anoxic conditions and can carry out anaerobic respiration, oxic and/or anoxic fermentation and can switch their metabolism (Rangel-Porras et al., 2019). Research into the ability of fungi to degrade OM under anoxic conditions has been expanding over the last decade within the deep biosphere (Ivarsson et al., 2016; Drake and Ivarsson, 2018; Zain UI Arifeen et al., 2019), groundwater (Perkins et al., 2019), and marine environments (Cathrine and Raghukumar, 2009; Lovelock et al., 2017). Certain fungi can function as facultative anaerobes (Ivarsson et al., 2016), using nitrate or nitrite as the terminal electron acceptor (Canfield et al., 2005), or as facultative chemolithotrophs, where metabolic energy is gained from sulfur oxidation (Xu et al., 2018). Given that sulfate reduction rapidly consumes oxygen during kelp degradation (Van Erk et al., 2020) and given that kelp is transported to anoxic/hypoxic environments (buried, or in the deep sea) (Krause-Jensen and Duarte, 2016), understanding oxygen limitation for a potentially facultative decomposing fungi is important for assessing the ability of fungi to contribute to kelp degradation.

The kelp genus *Ecklonia* occurs globally, and the species *Ecklonia radiata* is the principal macroalgal species in the temperate reefs of Australia (Kelaher et al., 2013). Here, we cultivated 11 endophytic fungi (10 individual species, with one species having 2 strains) from the brown macroalgal kelp species *E. radiata* and assessed their ability to degrade various organic polymeric compounds. Organic polymers can be used as a quick indicator for degradation potential and can identify microbial enzymatic activities, aiding to understand microbial degradation potentials (Caldwell et al., 2000; Promputtha et al., 2010; Perkins et al., *2*019) and the function of endophytes. We compared the polymeric organic matter decomposition abilities of these kelp endophytic fungi with four common terrestrial fungal species (3 Basidiomycota and 1 Ascomycota). To determine the ability of endophytic fungi to degrade kelp in low oxygen environments (buried in coastal sediments, or transported to the deep sea), we investigated organic polymer degradation indicated through dye decolorization under oxic and anoxic conditions for marine and terrestrial Ascomycota, Basidiomycota, and Mucoromycota. We hypothesized that (1) kelp endophytic fungi can degrade a variety of organic polymeric substrates under oxic conditions, (2) some kelp endophytic fungi can also degrade organic polymeric substrates under anoxic conditions, (3) the degradation potential under oxic and anoxic conditions differs between kelp endophyte species, and (4) there are functional differences between the degradation abilities of Ascomycota, Basidiomycota, and Mucoromycota. If kelp endophytic fungi that are symbiotic within living kelp have the capacity to degrade organic polymers, particularly under anoxic conditions, this suggests that they may become saprophytes of kelp detritus even when buried in the anoxic sediments, representing a currently unquantified coastal carbon cycling process.

## Experimental Procedures

### Endophytic Fungal Isolation and Cultivation

Whole and healthy (with no apparent symptoms of disease) *Ecklonia radiata* specimens were collected from water at 2–4 m depth in Charlesworth Bay, NSW, Australia (30°18′15″S, 153°9′5″E) then immediately transported in fresh seawater to the laboratory. The fresh kelp material was rinsed with sterile tap water and then blotted dry with autoclaved paper towels in a biosafety cabinet, equipped with a laminar flow-hood (Flewelling et al., 2013a). Surface sterilization was tested with 70% ethanol dip for varying durations (5, 10, 15, and 20s), and with Metrex cavicide (for the detailed surface sterilization method, please refer to Supplementary Material). A flame sterilized scalpel was used to cut the kelp material into discs for cultivation. For equipment sterilization, 70% ethanol was used.

From five surface-sterilized *E. radiata*, approximately 200 circular discs (5–8 mm diameter) were cut with a flame sterilized scalpel. These were taken from different sections (stipe, blade, and holdfast) of *E. radiata*, to represent the whole *E. radiata* organism. Discs from the various kelp sections were cultivated on separate plates. The discs were placed onto 70 Petri dishes (9 cm diameter, vented; 3 discs per Petri dish) containing either (1) 2% malt extract agar (MEA), (2) 2% MEA with sterilized seawater (MEA-SW), (3) selective agar for pathogenic fungi (SA) (Sigma Aldrich, BCBN8597TV), or (4) selective agar for pathogenic fungi with sterilized seawater (SA-SW). Seawater was collected during high tide at Seven Mile Beach (NSW, Australia 28.7906102S, 153.5940488E). These plates were incubated for up to 2 weeks at 22–24°C, and all emerging fungal endophytes were isolated onto 2% MEA. To prepare a stock library of each fungus, all isolates were further cultivated on 2% MEA.

### Molecular Identification of Fungal Isolates

To characterize each fungal isolate, ca. 250 mg DNA was extracted from the fungal mycelia of each isolate using the cetrimonium bromide (CTAB)-phenol-chloroform-isoamylalcohol/bead-beating protocol (Nercessian et al., 2005). Briefly, mycelial fragments were homogenized in a bead beater with zirconia/glass beads (0.1, 0.7, and 3 mm) and the following was added: (1) 600 μL 10% CTAB buffer in 1.6 M NaCl with a 1:1 ratio with 240 mM K_2_HPO_4_/KH_2_PO_4_ buffer, (2) 600 μL phenol:chloroform:isoamylalcohol (25:24:1), (3) 60 μL 10% N-lauryol sarcosine, and (4) 60 μL 10% sodium dodecyl sulfate (SDS). Following shaking (2,850 rpm for 10 min) and centrifugation (16,000× for 10 min at 4°C), the upper aqueous phase was removed and centrifuged in a new reaction tube with chloroform-isoamylacohol (24:1, 650 μL) to remove residual phenol. This solution was then incubated in 30% PEG 6000 prepared in 1.6 M NaCl (1.1 mL) to precipitate the DNA and was centrifuged again. The subsequent pellet was removed and cleaned with 1 mL icy 70% ethanol and centrifuged again. The ethanol was removed with a pipette, and the supernatant was dried at 37°C for up to 10 min. Finally, the recovered genetic material was dissolved in 50 μL PCR water and stored at −18°C until further processing.

For PCR, 2 μL of the stored DNA was mixed in 31.75 μL PCR water, 10 μL buffer, 2 μL bovine serum albumin (BSA), 1 μL each ITS1 and ITS4 (ITS1-5.8S-ITS2 region was amplified; White et al., 1990), 2 μL MgCl_2_ and 0.2 μL MyTaq Red DNA Polymerase (Bioline, Germany) and the PCR conditions were set at: (i) 95°C for 2 min, (ii) 35 cycles at 95°C for 30 s, (iii) 53°C for 30 s, (iv) 72°C for 45 s, and (v) 72°C for 5 s. The amplification was terminated at 10°C. PCR products were sequenced with Sanger technology at Macrogen Europe. Sequences were assembled using BioEdit (Hall, 1999). The taxonomic assignment of isolates was determined by comparing the ITS sequences against reference databases such as UNITE^1^ and the NCBI GenBank. Taxonomy was further curated using the Index fungorum.^2^ For the phylogenetic analysis, we aligned the sequences with MAFFT v7 (Katoh et al., 2018). We generated a maximum likelihood phylogenetic tree with FastTree version 2.1.9 (Price et al., 2010), using a GTR + G + I model of evolution. The resulting phylogenetic tree was displayed and edited in MEGA-X (Kumar et al., 2018). The fungal sequencing data was deposited to NCBI website (ID: 4751, MW999952-66). The link is included in Supplementary Material. For long term preservation, all isolates were freeze-dried at −80°C < 0.1 mBar (Labconco model 7934032) over 3 days.

### Organic Polymer Degradation Testing

For organic polymer degradation testing, the culture medium was prepared with, per liter, 0.94 g KH_2_PO_4_, 1.9 g K_2_HPO_4_, 1.6 g KCl, 1.43 g NaCl, 0.15 g NH_4_Cl, 0.037 g MgSO_4_, 0.1 g yeast, 10 g light-malt sugar, and 15 g agar. The solution was aliquoted into six sterile 1-liter Schott Duran glass bottles, kept in a water bath at 55°C, and supplemented with 100 mg streptomycin and penicillin to prevent bacterial contamination. To test for fungal organic polymer utilization, each of the following organic polymers were added to one of five aliquots: (**1)** 0.1% wt./vol 2,2’-Azino-bis(3-ethylbenzothiazoline-6-sulfonic acid) diammonium salt (ABTS), (**2)** 0.02% wt./vol Congo Red (CR), (**3)** 0.02% wt./vol Remazol Brilliant Blue (RBBR), (**4)** 0.02% wt./vol Toluidine Blue 199 (Tol), or (**5)** 0.02% wt./vol Bromocresol Green (Bromo). The degradation potential and/or bioactive compound production indicated by each of these substrates is detailed in Table 1. The sixth aliquot was used as a control, with no addition of any organic polymers allowing it to be compared to the growth of fungi with organic polymeric substrates.

**TABLE 1:** Isolated kelp endophytic fungi from *E. radiata* and their occurrence in different plant tissues(HF-holdfast, S-stipe, B-blade).

**ID**	**Species**	**Previously cultivated from other hosts/environments**	**Degradation potential and bioactive compounds**	**References**
1.	*Aspergillus proliferans* HF	Marine, terrestrial soil, in-door, citrus, grapevine	● Chitin degradation, ● Synthetic, or reactive dyes (aniline blue and congo red) degradation, Laccase enzyme production	Kanmani et al., 2011; Kim et al., 2013; Chen et al., 2017; Hassan et al., 2018
2.	*Bartalinia pondoensis* HF, S, B	Bamboo *Saraca asoca, Clerodendrum inerme, Phaseolus lunatus*	● Suppress salicylic acid, ● Anticancer activity, ● Detectable volatile compounds	Arzanlou, 2012; Navarro-Meléndez and Heil, 2014; Crous et al., 2015; Gong et al., 2015; Rashmi, 2019
3.	*Epicoccum sorghinum* HF, S	Air, soil *Dendrobium officinale* (stem and roots), *Camporesia sambuci*	● Tenuazonic acid production, ● Biocontrol agent against phytopathogens, ● Antimicrobial activity	Jin et al., 2017; Li et al., 2017; Braga et al., 2018; Oliveira et al., 2018
4.	*Cladosporium* sp. HF, S, B	Number of macroalgae	● Antioxidant activity ● Insecticidal activity	Flewelling et al., 2015
5.	*Talaromyces assiutensis* B	Soil, seawater, root halophytes, *Citrus macropter*	● Antimicrobial activity against *Staphylococcus epidermidis*, ● Anti-inflammatory, ● Anti-bacterial, ● Cyclopentenone derivative, phenolicethers derivative, itaconic acid derivative	Peter et al., 2012; You et al., 2014; Phonrod et al., 2016; Wang et al., 2016; Cai et al., 2020; Deka and Jha, 2020
6.	*Truncospora tephropora* HF, S	*Taxus chinensis, Centella asiatica*	● Anti-cancer compound ● *Pyricularia oryzae* activity, ● Sesquiterpenoid, perenniporin, ergosterol, decahydro-2,2,5,8-tetramethyl-2H-naphtho[1,8-bc]genfuran-5-ol, and albicanol, ● Cytotoxicitic metabolites, ● Alkaline resistant laccase production, ● Liquid waste detoxification	Ben Younes et al., 2007; Gómez-Toribio et al., 2009; Wu et al., 2013
7.	*Penicillium corylophilum* HF, S, B	Soil, waste water, *Alnus glutinosa* root, *Calotropis procera* leaf, *Picea glauca* needle, *Withania somnifera* stem	● Antibacterial activity, ● Glyceryl trinitrate (GTN), ● Organic matter removal and degrading hydrocarbons, ● Gibberellic acid production	Zhang et al., 1997; Garcia Silva et al., 2004; Nicoletti et al., 2014; Rai et al., 2014
8.	*Penicillium* sp. HF, S, B	Macroalgae	● Antioxidant activity ● Insecticidal activity	Flewelling et al., 2015
9.	*Penicillium fagi* HF, S, B	*Fagus silvatica*	● Water-soluble greenish blue pigments	Martinez and Ramirez, 1978; Mapari et al., 2005; Bouhri et al., 2020
10. 11.	*Mucor circinelloides* HF, S, B	Soil, plants and decaying fruits, *Vitis vinifera, Eichhornia crassipes, Alternaria porri*	● Biotechnology, ● Biodiesel production, ● Human health/fatal infection, ● Proteases production, ● Light sensing by three photoreceptor genes, ● Caratenogenesis	Abdel-Hafez et al., 2015; Zhao et al., 2016; Vellanki et al., 2018

*The table summarizes other environments from which the respective isolates have been cultivated previously and their known degradation potential. None of these species was present at marinefungi.org website.*

Once the aliquots were mixed, the media was poured into 9 cm diameter vented Petri dishes, and once solidified the media was inoculated with one of 11 cultivated kelp endophytic fungi or one out of four terrestrial fungi (one Ascomycota, cultivated from local soils, and three commercially available Basidiomycota [Oyster (*Pleurotus* sp.), Shiitake (*Lentinula* sp.), and Tasman Reishi (*Ganoderma* sp. from “Aussie mushroom supplies”), no strain number available)]. The total number of Petri dishes was 192 [**16** (15 fungi + 1 control) times **12** (5 organic polymer + 1 MEA control plates), duplicated for oxic and anoxic treatments]. To ensure that each Petri dish was inoculated with a similar mass of fungi, a flame sterilized molded wire was used. For each fungus, material for inoculations was taken from a single initial plate to ensure the same organism was used across treatments. The terrestrial fungi were chosen to gain a deeper knowledge of dissimilarities or similarities in degradation functions of the three fungal phyla and of kelp endophytic fungi from marine sources with common terrestrial fungi. Of the 192 plates, one set (96) was sealed with parafilm in front of the laminar flow hood and was packed into a clear still air box. To assess the degradation during anoxic conditions, the second set (96) was placed into a pre-sterilized anaerobic chamber. Any specimen transferred to the chamber was first purged with high purity N_2_. The anaerobic chamber was a COY model 10 gas analyzer, which automatically measures H_2_ and O_2_. Anoxic conditions were maintained at under 1–5% H_2_ in a N_2_ atmosphere at ambient temperature, using a Pd catalyst that consumes O_2_. This was monitored by an inbuilt alarm system that would be triggered if the gas concentration fell outside the chosen range. Due to the requirement for N_2_ purging, the second set of inoculates could only be sealed inside the anaerobic chamber. Any tool used in the chamber was first sterilized and purged with high purity N_2_. Kelp degradation where detritus was collected occurs at highly variable temperatures (in Australia 5–40°C); Petri dishes were kept at a median temperature (22–24°C) and under natural light conditions.

Each of the isolates was assessed weekly to determine growth and color change. Changes in color indicate catalytic conversion through the splitting of organic polymeric bonds in the substrates (Saroj and Narasimhulu, 2018). This enzymatic activity, known as hydrolysis, occurs through the production of acids or bases by the hydroxide anion and hydrogen cation of water during fungal OM degradation. The production of degrading enzymes changes the color of the media, and the type of enzymes determines the color change during hydrolysis (e.g., for CR loss of red color, or the media becoming clear would indicate acidic enzymatic production, whereas darkening of the media indicates alkaline enzymatic production). Hydrolysis was visually observed and was assessed (+, or ++) depending on the area of the specimen (diameter in cm) with changes in color to the media (Saroj and Narasimhulu, 2018) (if the color change did not exceed the area under the specimen received + and if the color change exceeded the area under the specimen received ++). Once a specimen received ++, or the specimen reached 90% growth on the Petri dish, it was kept for further observation but was not re-assessed. The isolates in oxic conditions were maintained for 6 weeks. As the anaerobic chamber acts as a laminar flow hood and as anaerobic degradation takes longer (Prasad et al., 2007), the isolates under anoxia were kept there for 16 weeks. After week 16, the fungi were transferred to a still air box and kept there for another week to observe any response following the change from anoxic to oxic conditions. Bacterial or mold contamination was not observed on any media throughout the experiments.

## Results

### Fungal Species

**FIGURE 1:**
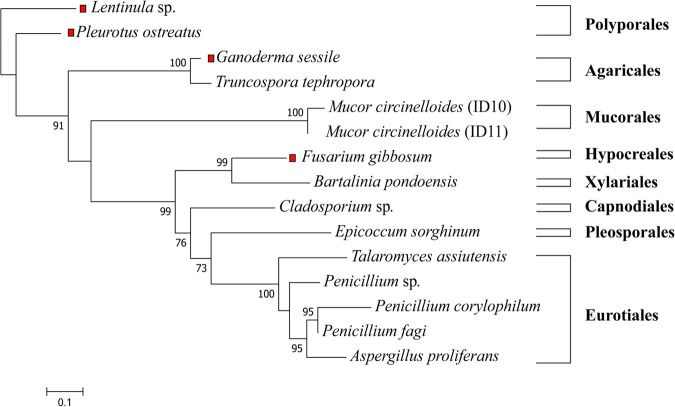
Maximum likelihood tree of all tested fungi in this study. The ITS1-5.8S-ITS2 sequences were aligned with MAFFT, the phylogenetic tree was generated with FastTree using a GTR + G + I model and visualized with MEGA X. Bootstrap values are indicated on the nodes. The red squares represent terrestrial fungi that were not derived from *E. radiata*. The scale represents the sequence divergence between the number of nucleotide substitutions per site.

Isolated strains were identified by visual morphological assessment (Supplementary Figure 1), and their identity confirmed via genetic analysis. A phylogenetic tree of the 11 fungal isolates from *E. radiata* kelp (Figure 1) assigned the fungi to three phyla (Ascomycota, Mucoromycota, and Basidiomycota), five classes (Eurotiomycetes, Sordariomycetes, Dothideomycetes, Mucoromycetes, and Agaricomycetes), six orders (Eurotiales, Xylariales, Pleosporales, Capnodiales, Mucorales, and Polyporales), and seven families (Aspergillaceae, Sporocadaceae, Didymellaceae, Trichocomaceae, Mucoraceae, and Polyporaceae) (Supplementary Table 2). From *E. radiata* tissues (blade, stipe, and holdfast), the species more frequently isolated were *M. circinelloides* (2 strains identified from 1 species on over 30 plates, although there may be other strains), *Penicillium* sp. (3 species on 23 plates, although there may be other species or strains), and *Cladosporium* sp. (15 plates). In contrast, *T. assiuntensis* was isolated only on four plates. Each plant section from *E. radiata* was separately cultivated to determine tissue specificity, particularly for the holdfast (Table 1).

### Organic Polymer Degradation

All *E. radiata* endophytic fungal isolates presented activities related with the degradation (change of color) of the polymeric compounds analyzed under oxic conditions, with varying effectiveness over time (Table 2 and Supplementary Table 1). CR is commonly used as a sensitive assay for the cellulase enzyme; this enzyme breaks down cellulose (Sazci et al., 1986; Florencio et al., 2012), which is the major component of *E. radiata* detritus. Strong decolorization of CR, which is associated with degradation of cellulose, was observed in *A. proliferans, E. sorghinum, Cladosporium* sp., *P. corylophilum, Penicillium* sp., *P. fagi*, and *M. circinelloides*, all of which produced significant (++) color change within 1 week. *G. sessile* and *T. tephropora* (endophytic Basidiomycota) changed the color of CR, but only by week three.

**TABLE 2:** Degradation of organic polymers by endophytic fungal isolates from kelp after 3 and 6 weeks under oxic conditions.

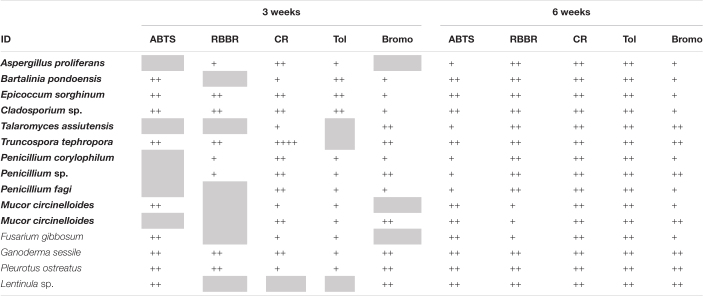

*Positive degradation activities are displayed using a semi-quantitative scale after visual interpretation of changes: blank, no growth; gray, growth but no hydrolysis; “+,” low hydrolysis ability; “++,” high hydrolysis ability. 2,2’-Azino-bis 3-ethylbenzothiazoline-6-sulfonic acid (ABTS) relates to laccase; Remazol Brilliant Blue R (RBBR) relates to lignin degradation; Congo Red (CR), Toluidine (Tol), and Bromocresol Green (Bromo) relates to triarylmethane and heterocyclic degradation, respectively. Isolates from E. radiata are in bold text.*

Hydrolysis (color change) of Tol is indicative of sulfur-containing lignin peroxidase activity (Hamza et al., 2012) and was observed within 1 week for *B. pondoensis, E. sorghinum, Cladsporium* sp. *A. proliferans, B. pondoensis, E. sorghinum, Cladosporium* sp., *P. fagi*, and *M. circinelloides.* No color change on Tol was detected for any Basidiomycota at this time and, overall, Basidiomycota showed a stronger color change of ABTS, RBBR, and Bromo than Ascomycota and Mucoromycota.

Within the first week, all Basidiomycota fully changed the color of ABTS, a compound associated with laccase activity (Leonowicz et al., 2001), whereas all Ascomycota required more time and showed less intensity in the color change. The change of color in RBBR by Basidiomycota, linked to ligninolytic and laccase activities (Sing et al., 2017), was slower than the color change on ABTS. Ascomycota and Mucoromycota showed a color change on RBBR only after 4 weeks. The color change of Bromo (associated with sulfonephthaleins lignin degrading enzymes) first occurred in week three for Basidiomycota, and in general, Ascomycota and Mucoromycota showed less activity. Overall, the *Fusarium* sp. patterns in changing the color of the media under oxic conditions were similar to Mucoromycota strains.

In general, growth or activity on polymers without any atmospheric oxygen was slower and no polymer changed color within the first 3 weeks (Table 3). CR was the first substrate to change color by week six under anoxic conditions; by this time four of the *E. radiata* endophytic fungal isolates (+) had changed the color of the media. *G. sessile* was the only Basidiomycota that changed color of any media, being ABTS by week 12. The control (MEA) was the least favored substrate for all fungi under anoxic conditions. The last 4 weeks of incubation resulted in more color changes for the same isolates. In contrast to Basidiomycota, endophytic Ascomycota successfully survived and revived after exposure to oxic conditions following 16 weeks of anoxia.

**TABLE 3:** Degradation of organic polymers by endophytic fungal isolates from kelp after 3, 6, and 12 weeks under anoxic conditions.

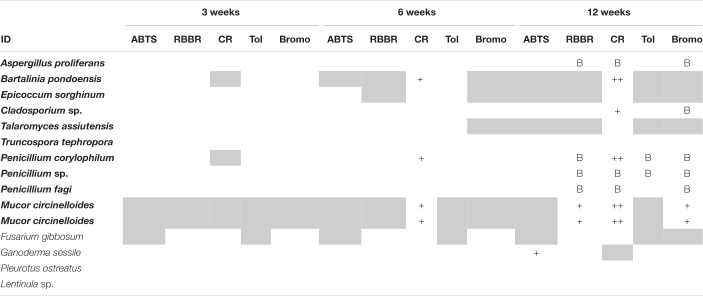

*Positive degradation activities are displayed using a semi-quantitative scale after visual interpretation of changes: blanks, no growth; gray, growth but no hydrolysis; “+,” low hydrolysis ability; “++”, high hydrolysis ability; B, visible breathing droplets on plates. 2,2’-Azino-bis 3-ethylbenzothiazoline-6-sulfonic acid (ABTS) relates to laccase; Remazol Brilliant Blue R (RBBR) relates to lignin degradation; Congo Red (CR), Toluidine (Tol), and Bromocresol Green (Bromo) relates to triarylmethane and heterocyclic degradation, respectively. Isolates from E. radiata are in bold text.*

## Discussion

### Community Composition of Kelp Endophytic Fungi

*E. radiata* had a diverse mycobiome of endophytic fungi and the 10 cultivated species and two strains represented three phyla: Ascomycota, Basidiomycota, and Mucoromycota (Supplementary Table 2). The phylum Ascomycota is the largest endophytic phylum (over 70,000 species in terrestrial and marine environments; Rashmi, 2019), and ca. 70% of endophytic fungi from *E. radiata* belong to this phylum. Eurotiomycetes, Dothideomycetes (Ascomycota), and Agaricomycetes (Basidiomycota) are the most commonly recorded endophyte classes (Rashmi, 2019) and were also dominant in *E. radiata* (eight of eleven isolates) (Supplementary Table 2). Pleosporales, Eurotiales, and Xylariales are the most commonly recorded endophytic orders (Rashmi, 2019), with seven of our eight isolates belonging to these orders (Supplementary Table 2). Here we cultivated five isolates (*Penicillium* sp., *Aspergillus* sp., and *Cladosporium* sp.; Supplementary Table 2), which are frequently isolated endophytes (Rashmi, 2019), from various macroalgae (Zuccaro et al., 2003, 2008; Suryanarayanan et al., 2010; Flewelling et al., 2013b; Sarasan et al., 2020). Endophytic fungi from *E. radiata* were cosmopolitan, found in other hosts or different environments (Table 1). Endophytic fungi are a diverse group, inhabiting and degrading terrestrial (Arnold, 2007; Kusari et al., 2012; Rana et al., 2019; Yan et al., 2019; Manganyi and Ateba, 2020) and marine vegetation (Hamzah et al., 2018; Noorjahan et al., 2019; Teixeira et al., 2019; Parthasarathy et al., 2020; Calado et al., 2021), linking ecology to plant pathology (Shipunov et al., 2008; Behie and Bidochka, 2014; Leach et al., 2017; Latz et al., 2018; Poveda et al., 2020), and evolution (Miyauchi et al., 2020; Tiwari and Bae, 2020), as well as plant communication and nutrient distribution (Kothe and Turnau, 2018; Sharifi and Ryu, 2021). Endophytic fungi live in symbiosis within the macroalgae, as in terrestrial vegetation (Jia et al., 2016), or in marine vegetation (Hamzah et al., 2018; Noorjahan et al., 2019; Calado et al., 2021) and are likely to regulate many biochemical pathways through their life cycle (Navarro-Meléndez and Heil, 2014; Latz et al., 2018). Clearly, endophytic fungi are likely of considerable importance, potentially for OM degradation (macrophytes, or other vegetation sources) and C cycling in terrestrial and marine environments.

### Organic Matter Degradation by Kelp Endophytic Fungi

This study focused on endophytic fungi from marine kelp, and we confirmed their presence. Our degradation assay demonstrates that kelp endophytic fungi can potentially turn to saprophytes, and we show that these fungi have the ability to decolorize several polymeric compounds found within the kelp detritus. Kelp detritus often travels great distances and our assay suggest that, after the death of *E. radiata* saprophytic endophytic fungi may be as important in marine sediments as has been reported for terrestrial soils (Mohamed and Martiny, 2011; Worden et al., 2015; Dini-Andreote et al., 2016; Ortega-Arbulú et al., 2019). Furthermore, endophytic fungi may influence kelp degradation in both oxic and anoxic environments (Cline et al., 2018; Wolfe and Ballhorn, 2020).

Fungal oxic degradation may be important in the water column, or on top of the kelp pile on the beach. The ability of the three phyla (Ascomycota, Basidiomycota, and Mucoromycota) to decolorize the various polymeric substrates differed significantly. Ascomycota and Mucoromycota primarily decolorized CR and Tol, which could indicate their cellulose degradation potential (pointing to the production of MnP, MiP, and sulfur-containing LiP), whereas Basidiomycota decolorized ABTS, RBBR, and Bromo (reflecting the potential production of laccase and LiP) (Supplementary Table 1). Importantly, although this study highlights the potential role of endophytic fungi in kelp degradation, the ability of fungi to degrade kelp detritus cannot be categorically determined through their dye decolorization abilities alone. However, the considerable differences in the dye decolorization ability of the various fungal species tested suggest that the decolorization was not simply caused by biosorption but was rather related to some difference in the biodegradation ability among the fungal species. These differences in degradation potential supports the ability of fungi to co-exist through niche-partitioning, as previously reported (Asgher et al., 2008). The composition of the fungal community within kelp may therefore be important for kelp detritus degradation.

Unlike in terrestrial environments, anoxic conditions are relatively common in coastal and marine sediments, where kelp detritus is deposited. The anoxic degradation potential of kelp endophytes is therefore particularly important. In this study we showed that some of the endophytic fungal isolates were able to decolorize CR in the absence of oxygen, indicating that they may have the potential to play a significant role in kelp (cellulose) degradation within anoxic sediments. This is in line with recent reports of anoxic fungal degradation in terrestrial (Ivarsson et al., 2016; Drake and Ivarsson, 2018) and marine environments (Barone et al., 2019; Zain UI Arifeen et al., 2019). CR was the substrate that was most decolorized by fungi under anoxia; this is not surprising, as CR is the most soluble and least recalcitrant of all substrates tested (Costa et al., 2012). Under oxic conditions the color change of CR media varied between species, from a loss in color to the media becoming dark (red, gray, or black) and, in some cases, later becoming losing color again after initially darkening. Under anoxic conditions, however, the media turned dark during hydrolysis. One single species of Basidiomycota (*G. sessile*) indicated activity on ABTS (associated with laccase production) under anoxic conditions. These results suggest that Ascomycota and Mucoromycota may have the potential to degrade cellulose under anoxic conditions.

Three endophytes were active under anoxia (*P. corylophilum*, *M. circinelloides*, and *B. pondoensis*) and of the isolated fungi, these three species showed the highest potential to degrade kelp in anoxic environments. The ability of these species to contribute to anoxic kelp degradation is not surprising. Under anoxic conditions *P. corylophilum* can reduce the chemical oxygen demand of activated sludge (Mannan et al., 2007), *B. pondoensis* produces cellulase (Yadav and Reddy, 2013) and emits volatile organic compounds (Navarro-Meléndez and Heil, 2014), and *M. circinelloides* has been shown to have the ability to use numerous C sources (e.g., xylose, arabinose, glycerol, starch, cellulose, or chitin) (Xia et al., 2011). *M. circinelloides* was the most active isolate under anoxic conditions, producing more cellulose-degrading and lignin-degrading enzymes (RBBR and Bromo). *M. circinelloides* is a yeast-mold dimorphic species, where dimorphism is influenced by C metabolism (Díaz-Pérez et al., 2020), thus it can switch between fermentative and oxidative processes (Crabtree effect—aerobic fermentation) (Rangel-Porras et al., 2019). Aerobic fermentation was found during macroalgae degradation (Van Erk et al., 2020). A likely pathway for fungi to break down macroalgal cellulose, whether in oxic or anoxic conditions, is via MnP or MiP production (Dos Santos et al., 2007) and subsequent splitting of cellulose bonds via hydrolysis (Conrad, 2006; Jung et al., 2015). MnP acts as a catalyst in the degradation of cellulose or phenolic and non-phenolic compounds in macroalgae, using H_2_O_2_ as an oxidant (Shin et al., 2005). The three endophytic fungi that were active under anoxic conditions in the current study probably work on charges alone and as such may not require oxygen. Aerobic fermentation during kelp degradation offers potential for more fungi to capitalize on the rich C source from kelp even in anoxic environments.

### Implications for Global Marine Carbon Cycling

The productivity of kelp is the highest amongst all coastal vegetation (Krause-Jensen and Duarte, 2016; Raven, 2018). Of all kelp, the widely distributed genus *Ecklonia* has moderately high productivity and detritus production (Krumhansl and Scheibling, 2012). Therefore, it is important to understand how C is cycled through the lifecycle *Ecklonia* sp., how that relates to marine carbon cycling, and to establish if the rich endophytic mycobiome has the ability to influence kelp degradation. In marine pelagic systems or coastal ecosystems, fungi have received far less attention than in terrestrial systems (∼1,900 documented marine species compared to over 100,000 documented terrestrial species) (Hyde et al., 1998; Ortega-Arbulú et al., 2019), and marine fungal degradation has received far less attention than marine bacterial OM degradation. Fungal pathways for kelp degradation are yet to be explored, whereas kelp degradation by bacteria (Bacteroidetes, Verrucomicrobia, Planctomycetes, Gammaproteobacteria, and Chlamydiae) has been intensively investigated (Sakai et al., 2003; Bengtsson et al., 2011; Silchenko et al., 2013; Sichert et al., 2020; Sun et al., 2021). Bacteria account for 60% (Sichert et al., 2020) to 90% (Koop et al., 1982) of kelp detritus degradation. Kelp detritus degradation by fungal degradation has been largely disregarded (Koop et al., 1982; Bengtsson et al., 2011; Van Erk et al., 2020), despite the dominant role of terrestrial fungi in refractory OM degradation (Gooday, 1990a; Tedersoo et al., 2014; Treseder and Lennon, 2015; Beaton et al., 2019). As has been reported previously, (Ivarsson et al., 2016; Drake and Ivarsson, 2018; Barone et al., 2019) the current study demonstrates that, like bacteria, some fungi may be active under anoxic conditions and have the capacity to break down kelp cellulose in various habitats. In terrestrial environments, an antagonistic interaction between bacteria and fungi is reported (Gooday, 1990b; Mille-Lindblom and Tranvik, 2003), and this may also be true for the marine environment. As cellulose is the most abundant polymer in marine systems, cellulose degradation is an important process for global C cycling (Leschine, 1995; Hyde et al., 1998; Worden et al., 2015; Ortega-Arbulú et al., 2019; Chrismas and Cunliffe, 2020). The role of both fungi and bacteria for kelp detritus degradation processes needs to be evaluated in further detail. Given that fungal polymeric OM degradation in terrestrial systems outcompetes bacterial degradation (Malik et al., 2016; Fabian et al., 2017), fungi in anoxic marine sediments may also contribute significantly to global C cycling (Worden et al., 2015; Lakshmi et al., 2017; Balabanova et al., 2018).

### Future Research

To date, kelp degradation by fungi has received little attention and this study highlights that endophytic fungi in kelp have the potential to contribute to kelp detritus degradation. Cultivation based techniques would be complemented with further studies; DNA analysis to confirm the endophytic fungal community within the macroalgae and genomics to confirm the specific role of fungi in kelp detritus degradation with the underlying mechanisms, such as the relative contribution of specific enzymes (e.g., MnP, MiP, and LiP). Kelp degradation can be assessed by using a single dye or a mixture of dyes coupled with different environmental factors (e.g., pH, temperature, oxygen, and agitation) (Rani et al., 2014; Sen et al., 2016). Biosorption is a major mechanism in dye decolorization and can address the differences (e.g., deposition, entrapment in inner spaces, surface ion-exchange, complexation, precipitation, adsorption, and the formation of hydrogen bonds) within living and dead fungal cells (Yeddou-Mezenner, 2010). Moreover, coastal contamination (e.g., agriculture, or waste processing) can influence macroalgal community dynamics and seedling bioassays using different dyes can define how these contaminants affect growth rate, or the physiological fitness of the macroalgal community. Pollution also holds the potential to impact the kelp endophytic fungal community, their kelp degradation rates and thus marine C cycling.

## Data Availability Statement

The datasets presented in this study can be found in online repositories. The names of the repository/repositories and accession number(s) can be found below: https://www.ncbi.nlm.nih.gov/, ID: 4751, MW999952-66.

## Author Contributions

AP helped conceive the project, collected, analyzed, and interpreted the data, and drafted the manuscript. AR, H-PG, and JO helped conceive the project and contributed substantially to the manuscript. KR-J contributed substantially to data analysis and interpretation. SB contributed to collecting specimens and data and assisted with the manuscript. All listed authors have contributed substantially to the preparation and drafting of this manuscript and have approved the final submitted manuscript.

## Conflict of Interest

The authors declare that the research was conducted in the absence of any commercial or financial relationships that could be construed as a potential conflict of interest.

## Publisher’s Note

All claims expressed in this article are solely those of the authors and do not necessarily represent those of their affiliated organizations, or those of the publisher, the editors and the reviewers. Any product that may be evaluated in this article, or claim that may be made by its manufacturer, is not guaranteed or endorsed by the publisher.

## Footnotes

1
https://unite.ut.ee/analysis.php

2
http://www.indexfungorum.org/names/names.asp
