# Supplementary material for: Oxic and Anoxic Organic Polymer Degradation Potential of Endophytic Fungi From the Marine Macroalga, Ecklonia radiata
Source: Front Microbiol. 2021 Oct 18;12:726138. doi: 10.3389/fmicb.2021.726138 (PMC8558676; doi:10.3389/fmicb.2021.726138)
Supplement: Supplementary file 1 [file Data_Sheet_1.docx]

**Oxic and anoxic organic polymer degradation potential of endophytic fungi from the marine macroalga, *Ecklonia radiata***

**Anita K. Perkins^1,2^, Andrew L. Rose^1,2^, Hans-Peter Grossart^3,4^, Keilor Rojas-Jimenez^5^, Selva K. Barroso Prescott^6^, Joanne M. Oakes^1^**

^1^ Centre for Coastal Biogeochemistry, Faculty of Science and Engineering, Southern Cross University, Lismore, 2480 NSW, Australia

^2^ Southern Cross Geoscience, Faculty of Science and Engineering, Southern Cross University, Lismore, NSW, Australia

^3^Leibniz Institute for Freshwater Ecology and Inland Fisheries (IGB), Experimental Limnology, 16775 Neuglobsow, Germany

^4^University of Potsdam, Institute of Biochemistry and Biology, Maulbeerallee 2, 14469 Potsdam, Germany

^5^ Escuela de Biologia, Universidad de Costa Rica, 11501, San Jose, Costa Rica

^6^ National Marine Science Centre, Faculty of Science and Engineering, Coffs Harbour, NSW, Australia

*** Correspondence:**Hans-Peter Grossart
hgrossart@igb-berlin.de

**Keywords:** **Kelp_1_, fungi_2_, endophytes_3_, carbon cycling_4_, extracellular enzymes_5_, cellulose_6_**

# **1 Additional Information on Experimental Procedures**

## **1.1 Surface sterilisation method for endophytic fungal isolation**

Surface sterilisation is an important step that must be carried out when cultivating endophytic fungi (Hyde and Soytong, 2008). A variety of surface sterilisation techniques were tested to remove potentially contaminating ectobionts from the surface of *E. radiata*; these methods included: 1) submerging samples of *E. radiata* into 70% ethanol for 5, 10, 15 or 20 seconds (Suryanarayanan *et al.*, 2010; Flewelling *et al.*, 2013), and 2) wiping the outer surface of *E. radiata* samples with autoclaved paper towel using 70% ethanol and also with metrex cavicide, with or without subsequent rinsing with sterile seawater to remove ethanol and metrex cavicide. Metrex cavicide is a known surface laboratory equipment steriliser and to our knowledge was not previously tested during cultivation. Two methods of disc cutting techniques were tested: 1) discs that were individually sterilised, and 2) larger pieces of *E. radiata* that were sterilised, from which only the inner discs were used. To determine the effectiveness of each sterilisation technique, 3 plant discs were sterilised using each method then placed onto an agar plate made up of 2% malt extract agar (MEA), and 2% MEA with sterilised seawater. Seawater was used as most marine isolates grow better with the addition of seawater (Pawar and Thirumalachar, 1966; Zhang *et al.*, 2015). The aim was to remove ectobionts, whilst allowing cultivation of endophytic fungi.

Surface sterilisation of larger *E. radiata* pieces seemed more effective, where discs were cut from the middle of the sterilised piece. With the larger sterilised pieces, the inner fungi was less impacted by the sterilisation and meant that species grew faster with less ectobionts present. The 20 s ethanol dip and the ethanol/metrex cavicide wipe, prevented any growth of ectobionts or endophytic fungi for over 30 days, the 15s ethanol dip removed ectobionts and allowed endophytic fungal growth. However, the growth of endophytes following 15s in ethanol was extremely slow. Submerging kelp material in 70% ethanol for 10s removed ectobionts whilst allowing for good cultivation of endophytic fungi. The 5s submerging was not sufficient to kill enough ectobionts, thus the 10s submerging surface sterilisation method was used throughout the study.

# **1.2 Data Availability Statement**

The fungal sequencing datasets for this study can be found in the [NCBI, ID: 4751, MW999952-66] [<https://www.ncbi.nlm.nih.gov/nuccore/?term=Oxic+and+anoxic+organic+polymer+degradation+by+endophytic+fungi+from+marine+macroalgae>].

## **1.2 Organic polymer testing**

**Table S1.** Organic polymeric compounds used in this study, their features, and the enzymatic pathways they indicate.

| **Organic polymer** | **Chemical compounds** | **Enzymatic pathways** | **Features** |
| --- | --- | --- | --- |
| ABTS | 2,2’-azino-bis(3-ethylthiazoline-6-sulfonate, (C_18_H_18_N_4_O_6_S_4_) | Laccase activity, lignin degradation, demethylation, quinone formation | The copper-containing laccase produced by fungi (benzenediol: oxygen oxidoreductase) oxidizes a range of aromatic hydrogen donors by electron/proton removal, forming phenoxy radicals, or amino radicals and also can decarboxylate through demethylation (Leonowicz et al. 2001). Laccases oxidize aromatic hydrogen donors and catalyses the electron and proton removal from phenolic hydroxyl, or phenoxy radicals, forming aromatic amino groups (Leonowicz et al. 2001). |
| Congo Red | [3,3'-(Biphenyl-4,4'-diyldidiazene-2,1-diyl)bis(4-aminonaphthalene-1-sulfonic acid))](https://pubchem.ncbi.nlm.nih.gov/compound/11314), (C_32_H_22_N_6_Na_2_O_6_S_2_) | Cellulose and chitin degradation, lignin peroxidase and manganese-dependent peroxidase activity | Disodium salt diazodye that is more soluble than ethanol. It possesses a strong, non-covalent bond but has high affinity to cellulose and it is also used as a pH indicator as the solution turns red in alkaline conditions and blue in acid conditions. |
| Remazol Brilliant Blue R (RBBR) | [1-Amino-9,10-dioxo-4-[[3-(2-sulfooxyethylsulfonyl)phenyl]amino]anthracene-2-sulfonic acid)](https://pubchem.ncbi.nlm.nih.gov/compound/17410) (C_22_H_16_N_2_Na_2_O_11_S_3_) | Lignin degradation and laccase activity | Anthraquinone based vinylsulphone dye (Sing et al. 2017) for ligninolytic (Novotnýeněk et al. 2001) and laccase activity but the delignification is not as efficient as ABTS (Leonowicz et al. 2001). This anthracene derivative organic pollutant is soluble in water but insoluble in ethanol (Hadibarata et al. 2012). |
| **Organic polymer** | **Chemical compounds** | **Enzymatic pathways** | **Features** |
| Toloudine blue (Tol) | 3-amino-7-(dimethylamino)-2-methylphenothiazin-5-ium) heterocyclic dye, (C_15_H_16_ClN_3_S ) | Lignin and cellulose degradation with sulphur containing lignin peroxidase | Also known as tolonium chloride, is a heterocyclic dye with redox properties (Harith et al. 2014). It is an acidophilic methachromatic dye in the phenothiazine group that selectively stains sulphates, carboxylates and phosphate (Sridharan and Shankar, 2012). It stains cellulose with blue and lignin with green (Nagamoto et al. 2011). Partially soluble in water and ethanol. Tol as a substrate is oxidised by nitrite ions with a hydrogen ion dependence and indicates nitrification processes with 3:1 mole ratio to Tol to nitrite ions (Hamza et al. 2012). |
| Bromocresol Green (Bromo) | 3,3-Bis(3,5-dibromo-4-hydroxy-2-methylphenyl)-3H-benzo[c][1,2]oxathiole 1,1-dioxide) triphenylmethane dye, (C_21_H_14_Br_4_O_5_S) | Sulfonephthaleins class indicating lignin peroxidase activity | Aromatic sodium salt that is a heterocyclic, triphenylmethane phenol dye belonging to the sulfonephthaleins class, with hydroxyl groups has two aryl groups at *p* position (*Bromocresol green \| C21H14Br4O5S - PubChem*). Sparingly soluble and it is also used as a pH indicator. At pH 3.8 (monoanionic form) it turns yellow indicating citric acid production (Auta et al. 2014), whereas at higher pH (dianionic form) when indicating ionized enzymatic production it turns green (pH 5.4) to blue (pH 7). |

# **2 Additional Information on Results**

**Table S2.** Genetically confirmed taxonomy for each fungus examined in the degradation experiments*.* Isolates from *E. radiata* are shaded grey

| **ID** | **Phylum** | **Class** | **Order** | **Family** | **Genus** | **Species** | |
| --- | --- | --- | --- | --- | --- | --- | --- |
| 1 | Ascomycota | Eurotiomycetes | Eurotiales | Aspergillaceae | *Aspergillus* | *Aspergillus proliferans* | |
| 2 | Ascomycota | Sordariomycetes | Xylariales | Sporocadaceae | *Bartalinia* | *Bartalinia pondoensis* | |
| 3 | Ascomycota | Dothideomycetes | Pleosporales | Didymellaceae | *Epicoccum* | *Epicoccum sorghinum* | |
| 4 | Ascomycota | Dothideomycetes | Capnodiales | Cladosporiaceae | *Cladosporium* | *Cladosporium sp.* | |
| 5 | Ascomycota | Eurotiomycetes | Eurotiales | Trichocomaceae | *Talaromyces* | *Talaromyces assiutensis* | |
| 6 | Basidiomycota | Agaricomycetes | Polyporales | Polyporaceae | *Truncospora* | *Truncospora tephropora* | |
| 7 | Ascomycota | Eurotiomycetes | Eurotiales | Aspergillaceae | *Penicillium* | *Penicillium corylophilum* | |
| 8 | Ascomycota | Eurotiomycetes | Eurotiales | Aspergillaceae | *Penicillium* | *Penicillium sp.* | |
| 9 | Ascomycota | Eurotiomycetes | Eurotiales | Aspergillaceae | *Penicillium* | *Penicillium fagi* | |
| 10 | Mucoromycota | Mucoromycetes | Mucorales | Mucoraceae | *Mucor* | *Mucor circinelloides* | |
| 11 | Mucoromycota | Mucoromycetes | Mucorales | Mucoraceae | *Mucor* | *Mucor circinelloides* | |
| 12 | Ascomycota | Sordariomycetes | Hypocreales | Nectriaceae | *Fusarium* | *Fusarium gibbosum* | |
| 13 | Basidiomycota | Agaricomycetes | Polyporales | Polyporaceae | *Ganoderma* | *Ganoderma sessile* | |
| 14 | Basidiomycota | Agaricomycetes | Agaricales | Pleurotaceae | *Pleurotus* | *Pleurotus ostreatus* | |
| 15 | Basidiomycota | Agaricomycetes | Polyporales | Polyporaceae | *Lentinula* | *Lentinula* sp. |  |


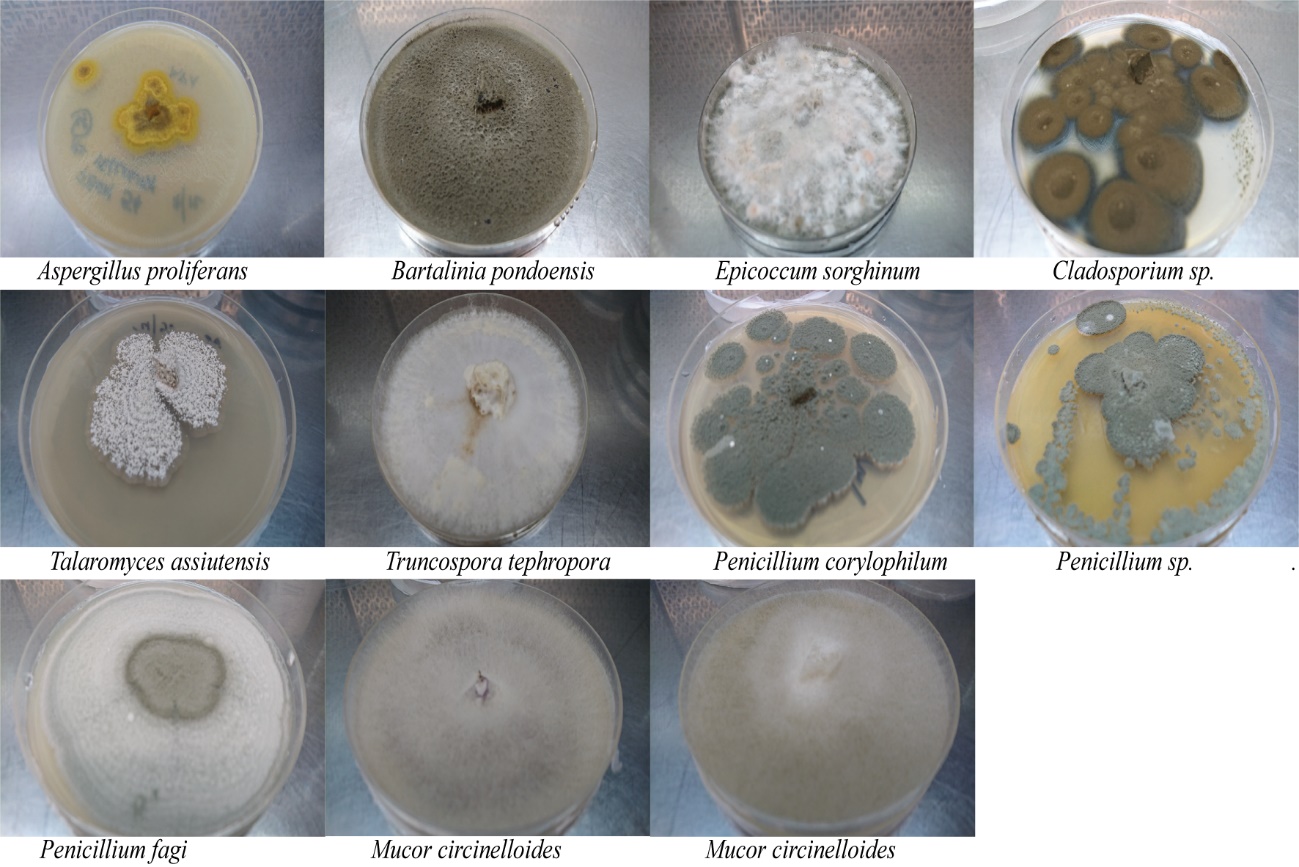


**Figure S1.** Images from cultivated endophytic kelp fungi

References

Auta, H. S., Abidoye, K. T., Tahir, H., Ibrahim, A. D., & Aransiola, S. A. (2014) Citric Acid Production by *Aspergillus niger* Cultivated on Parkia biglobosa Fruit Pulp . *Int Sch Res Notices*. doi: 10.1155/2014/762021

Hadibarata, T., Yusoff, A. R. M., & Kristanti, R. A. (2012) Decolorization and metabolism of anthraquionone-type dye by laccase of white-rot fungi polyporus sp. S133. *Water, Air, and Soil Pollut*. doi: 10.1007/s11270-011-0914-6

Harith, Z. T., Ibrahim, N. A., & Yusoff, N. (2014) Isolation and identification of locally

isolated lignin degrading bacteria. *J Sustain Sci Manage.* 9 (2) 114-118

Hamzah, T. N. T., Lee, S. Y., Hidayat, A., Terhem, R., Faridah-Hanum, I., and Mohamed, R.

(2018) Diversity and Characterization of Endophytic Fungi Isolated From the

Tropical Mangrove Species, *Rhizophora mucronata*, and Identification of Potential

Antagonists Against the Soil-Borne Fungus, *Fusarium solani*. *Front Microbiol*.

0:1707. doi: 10.3389/fmicb.2018.01707

Leonowicz, A. et al*.* (2001) Fungal laccase: Properties and activity on lignin. *Basic*

*Microbiol*. 41 (3-4) 185–27. doi: 10.1002/1521-4028(200107)

Nagamoto, N. S., Garcia, M. G., Forti, L. C., Verza, S. S., Noronha, N. C., & Rodella, R. A. (2011) Microscopic evidence supports the hypothesis of high cellulose degradation capacity by the symbiotic fungus of leaf-cutting ants. *J Biol Res*. 16. 308-312

Novotnýeněk, Rawal, B., Bhatt, M., Patel, M., Šašek, V., & Molitoris, H. P. (2001). Capacity

of Irpex lacteus and *Pleurotus ostreatus* for decolorization of chemically different

dyes. *J Biotechnol.* doi: 10.1016/S0168-1656(01)00321-2

Sing, N. N., Husaini, A., Zulkharnain, A., & Roslan, H. A. (2017). Decolourisation

Capabilities of Ligninolytic Enzymes Produced by *Marasmius cladophyllus* UMAS

MS8 on Remazol Brilliant Blue R and Other Azo Dyes. *BioMed Res Int*. doi:

10.1155/2017/1325754

Sridharan, G., & Shankar, A. A. (2012) Toluidine blue: A review of its chemistry and clinical utility. *Journal of Oral and Maxillofac Pathol.*doi: 10.4103/0973-029X.99081
